# Supplementary material for: MetaRibo-Seq measures translation in microbiomes
Source: Nat Commun. 2020 Jun 29;11:3268. doi: 10.1038/s41467-020-17081-z (PMC7324362; doi:10.1038/s41467-020-17081-z)
Supplement: Supplementary file 10 — Supplementary Data 7 [file 41467_2020_17081_MOESM10_ESM.zip › File2/Confidence_High_Taxonomy/183458_out.krona.html]

Javascript must be enabled to view this page.

members
magnitude
magnitudeUnassigned
count
unassigned
taxon
rank

183458\_out

7

7
2
superkingdom

7
1239

SRS042284\_contig\_number\_contig-100\_2963.2963SRS148159\_contig\_number\_5746
phylum
2

class
186801
5

order
186802
5

family
5
186806

5
1730
genus
3

SRS017521\_contig\_number\_11641SRS098717\_contig\_number\_3469SRS1054691\_contig\_number\_2612


SRS019601\_contig\_number\_12499
457402
1
species

species

SRS146888\_contig\_number\_9918
2302950
1
